# The Provincial Medical and Surgical Association

**Published:** 1838-07

**Authors:** 


### THE PROVINCIAL MEDICAL AND SURGICAL ASSOCIATION.

The steady progress of the affairs of this Association, from the period of its institution, six years ago, to the present time, serves most undeniably to evince that its founders made a correct estimate of the existing desire among the cultivators of medical science residing in the English provinces to combine together, so as to make their labours more instrumental than they had formerly been in the advancement of the healing art. Every anniversary has shown a great increase of members; and the influence which has arisen from this additional strength has been directed to the accomplishment of the important objects which were, at the commencement of the undertaking, specified as most worthy of regard: consequently, there can be little doubt that each returning year will afford fresh proofs of the advantages that must result from the continuance of an Association which has in view the promotion of harmony and good feeling among its members, and the collection and diffusion of facts which may serve to render medical science more signally beneficial to mankind.

The members now amount to at least eleven hundred, and the number is progressively increasing. The happy project of establishing *District Branches* has been a fruitful source of the Association's prosperity, and promises to extend its benefits, in a very short time, over the whole kingdom. In addition to the Branches formerly noticed, two new ones have been formed during the last and present year; viz. one at Newton, in Lancashire, called the *Newton Branch*, comprehending members residing in Manchester, Liverpool, Warrington, and several other towns in the north of England; the other at Shrewsbury, called the *Shropshire and North Wales Branch.* These branches, in imitation of the parent Society, hold local and annual meetings for the discussion of medical subjects, reading reports on the progress of medicine and surgery, and watching over and promoting the interests of the profession generally. The place of the annual meetings of the Branch-Societies, like that of their parent, is changed on every occasion, for the convenience of the members. Thus, the *Southern Branch* held its general meeting last year at Winchester, this year at Salisbury, and it will hold it next year at Portsmouth. The meeting at Salisbury took place on the 14th of last month, and was attended by from sixty to eighty members. Dr. Fowler was president. Much valuable matter was brought forward, which led to many interesting discussions. The members afterwards dined together. On this occasion, a prize of twenty guineas was announced for the best essay on Vaccination, the gift of one of the members, Mr. Newnham, of Farnham. The essays are to be transmitted to the secretary three months before the annual meeting in June, 1840. Another prize, of fifty pounds, was offered last year, at the Cheltenham meeting of the Parent Association, by Dr. Thackeray, of Chester, for the best essay having for its object the investigation of the sources of the common Continued Fevers of Great Britain and Ireland, and the ascertaining of the circumstances which favour the diffusion of these diseases, and also those circumstances which may have a tendency to render them communicable from one person to another. The prize, like Mr. Newnham's, will be open to the competition of the members of every accredited school for medicine and surgery in the united kingdom, and the essays are to be sent to the secretaries of the Association on or before the 1st of January, 1840.

The annual meeting of the Association takes place this year at Bath, on the 18th and 19th of this month (July), under the presidency of Dr. Barlow. A very large reunion of members is expected; and it is understood that many topics of great interest will be discussed; among others, the present state of vaccination, and the best means of putting a stop to the lamentable ravages of small-pox. The Retrospective Address will be delivered by Dr. Malden, of Worcester.

